# Metropolitan Hospital Sunday Fund

**Published:** 1903-08-08

**Authors:** 


### HOSPITAL ADMINISTRATION.

### CONSTRUCTION AND ECONOMICS.

### METROPOLITAN HOSPITAL SUNDAY FUND.

#### I.—ITS NEW DEPARTURE.

THE report of the Distribution Committee of the Hospital Sunday Fund, which will be found in another column, is unique in two respects, so far as this Fund is concerned. In the first place, it records the visit of their Majesties the King and Queen to St. Paul's Cathedral on the afternoon of Sunday, June 7th, in support of Hospital Sunday, when the collections amounted to £5,500, which is probably the largest sum ever raised in a place of worship on behalf of the hospitals. We believe that this spontaneous act on the part of the King and Queen is well calculated to give a stimulus to the Hospital Sunday movement throughout the country, and that every community like that of the metropolis of the Empire, where despite the drenching wet, which made the day notable in the annals of wet Sundays, the sum raised has proved to be a record, must be aroused to a special effort through this movement in the cause of their sick and suffering neighbours. The second important feature of the report are the statements in it that the Committee have not included this year the outlay on repairs and improvements of buildings in arriving at their basis of award, and they have further directed special attention to the constant increase in the expenditure of hospitals on the maintenance of patients, and have reduced the basis of award in no less than 38 cases. We take it that the effect of this last step is calculated to arouse much attention and discussion, and we have therefore thought it well to analyse the awards in bulk and in detail, in order that the effects of this new departure on the part of the Distribution Committee may be exactly recorded.

First of all it must be borne in mind that the Committee have distributed this year £58,386, being £578 more than last year, when the sum they had to apportion was only £57,808. The following table shows the groups of hospitals and institutions which have received awards. In the first column, after the name of the class of institution, will be found two sets of figures, and in each case the first represents the number of institutions receiving grants in 1902, and the second those receiving them in 1903. It will be seen further that every group of institution received increased grants during 1903 with the exception of the general hospitals, the hospitals for women, and the dispensaries. Of course certain individual hospitals in each of the three latter groups, as will be seen in subsequent tables, have received larger grants in 1903 than they did in 1902, though collectively the total assigned to their section was less this year than last.

AWARDS OF THE DISTRIBUTION COMMITTEE 1902 AND 1903.

This table gives an analysis of the amounts given collectively to each group of institution represented in the classification of the Distribution Committee in 1903 compared with 1902. It also shows the actual increase or decrease in figures and the approximate percentage of gain or loss for the year.

| Class of Institution. | No. of Beds. | | Total Awards. | | Increase. | Percentage of Increase. | Decrease. | Percentage of Decrease. |
|---|---|---|---|---|---|---|---|---|
| | 1902 | 1903 | 1902 | 1903 | | | | |
| | | | £ | £ | £ | | £ | |
| General Hospitals.. | 31 | 31 | 33.202 | 32,341 | | | −861 | −2·6 |
| Chest ,, | 5 | 5 | 4,217 | 4 485 | +268 | + 5·5 | — | — |
| Children's ,, | 17 | 17 | 4,356 | 4.588 | +232 | + 5·06 | — | — |
| Lying-in ,, | 5 | 6 | 680 | 723 | + 43 | + 6· | — | — |
| Women ,, | 6 | 6 | 1.805 | 1.652 | | | −153 | −8 48 |
| Special ,, | 27 | 28 | 5,946 | 6,359 | +413 | + 6·49 | — | — |
| Convalescent .. | 28 | 30 | 3,280 | 3.308 | + 28 | + 85 | — | — |
| Cottage .. .. | 18 | 20 | 1.069 | 1,433 | +364 | +25·4 | — | — |
| Various Institutions | 11 | 12 | 872 | 1.278 | +406 | +31·8 | — | — |
| Dispensaries .. | 56 | 56 | 2,381 | 2 219 | | | −162 | −6·8 |
| | 204 | 211 | 57.808 | 58,386 | 1,754 | 81·1 | 1,176 | 17 88 |

Net increase = £578.

Taking the table in detail it will be noticed that, whereas the small charities of various kinds classified as "institutions," amounting in all to twelve, have had the money apportioned to them increased to the extent of 31·8 per cent., that is by £406, and the cottage hospitals, of which twenty receive awards, have had their grants increased by 25·4 per cent., *i.e.* by £364, the great general hospitals have actually received 2·6 per cent. less in 1903 than they received in 1902, or, in other words, £861 of the money which was apportioned to them in 1902, when the total sum distributed was £578 less than in the present year, has been taken out of their pockets and put into the pockets of other institutions in the proportions shown in the table. Hospitals for diseases of the chest have received 5½ per cent. more; hospitals for children have received 5 per cent. more; lying-in hospitals, 6 per cent. more; special hospitals, 6½ per cent. more; and the convalescent institutions between ¾ and 1 per cent. more in 1903 than they received last year. On the other hand, the hospitals for women have received 8½ per cent. less, and the dispensaries nearly 7 per cent. less in 1903 than in 1902. We think that the diminution in the grant to the dispensaries is greatly to be regretted, and we have thought it well to reduce the awards to precise figures, so as to reveal the working of the new rule as it stands at present, because we hope that in future years the Distribution Committee may be able to so adjust matters as to provide that the general hospitals and the dispensaries are not again made to contribute directly to an increased grant to practically all other institutions with which the Distribution Committee have to deal. We express this hope because we cannot believe that either the needs and merits or the actual circumstances as they exist to-day in regard to all the London hospitals and medical charities can be shown to warrant the deduction of over £1,000 from the grants made to the general hospitals and dispensaries by the Sunday Fund in 1903, and the withholding from them of any portion of the increased money available (£578) to the advantage of the remaining institutions which have been fortunate enough to win the suffrages of the Distribution Committee.

*(To be continued.)*

